# International approaches to orthopaedic sports injury education: Advancing clinical practice through global collaboration

**DOI:** 10.1016/j.jcot.2025.103178

**Published:** 2025-08-15

**Authors:** Filippo Migliorini, Ludovico Lucenti, Michele Mercurio, Leonardo Puddu, Tommaso Bardazzi, Jörg Eschweiler

**Affiliations:** a Department of Trauma and Reconstructive Surgery, University Hospital of Halle, Martin-Luther University Halle-Wittenberg, 06097 Halle (Saale), Germany; b Department of Orthopaedic and Trauma Surgery, Academic Hospital of Bolzano (SABES-ASDAA), via Lorenz Böhler 5, 39100, Bolzano, Italy; c Department of Life Sciences, Health, and Health Professions, Link Campus University, Via del Casale di San Pio V, 00165 Rome, Italy; d Department of Precision Medicine in Medical, Surgical and Critical Care (Me.Pre.C.C.), University of Palermo, 90133 Palermo, Italy; e Orthopaedic and Traumatology Department, Santa Maria del Carmine Hospital, 38068 Rovereto, Italy; f Department of Orthopaedic and Trauma Surgery, Dulbecco University Hospital of Catanzaro, Magna Graecia University, Italy

**Keywords:** Orthopaedic training, Simulation-based learning, Clinical competence, Surgical education, Global disparities, Multidisciplinary approach, Digital platforms

## Abstract

Sports injuries represent a growing global health challenge, placing substantial demands on healthcare systems and requiring multidisciplinary management. As orthopaedic sports trauma becomes increasingly complex, high-quality education and standardised training are essential to improve prevention, diagnosis, and treatment outcomes. This narrative review aims to analyse current educational models and training pathways in orthopaedic sports injury management worldwide, evaluate the role of technological innovations, and highlight opportunities for international collaboration to enhance clinical practice and education. A wide spectrum of training methods is currently employed, ranging from traditional time-based models to modern competency-based curricula. Innovations such as virtual and augmented reality, simulation-based learning, and e-learning have demonstrated promising results in surgical skill acquisition and knowledge retention. International fellowships and consensus frameworks further contribute to the harmonisation of training standards. However, disparities persist in access to educational resources, healthcare infrastructure, and implementation of new technologies, particularly in low- and middle-income countries. To meet the demands of contemporary orthopaedic sports medicine, educational strategies must evolve through global collaboration, integration of emerging technologies, and commitment to equity. Standardised, multidisciplinary, and evidence-informed training models are needed to ensure optimal patient care and professional competence across diverse healthcare environments.

## 1 Introduction

Sports injuries involve a significant expenditure of hospital and human resources. Sports injuries often lead to emergency room visits and require specialist, surgical, and rehabilitative treatment. Approximately 18–30 % of all acute injuries are sports-related.^1^, ^2^, ^3^, ^4^ Worldwide increased involvement in sports and physical activities leads to an increase in sports injuries, resulting in a growing demand for highly specialised orthopaedic treatment.^4^, ^5^, ^6^ Sports-related injuries are gaining attention lately, not only because of the possible short- and long-term consequences for athletes but also because of the economic burden these injuries represent. Orthopaedic fellowships gained popularity in the United States in the 1970s, and since then, the number of orthopaedic residents who pursue subspecialty training has increased. Previous studies have shown a rise in subspecialties from 1988 to 2002.^7^ In the USA, 90 % of Orthopaedic surgeons do a fellowship, mostly in Sports Medicine.^8^ Following the example of other areas in general traumatology, where specialists from various countries have established collaborative networks to improve and standardise care, there is a need for orthopaedic education and training programs that specifically focus on the management of sports injuries. Furthermore, an overemphasis on the volume of individual surgeon cases fosters a training environment where technical skills are prioritised and can distract from teaching the fundamental principles of medical practice, including shared decision-making. Therefore, developing standardised programs is essential for clinical practice and ensuring the best results for patients. Medical practice variation in the United States results in higher costs without corresponding improvements in patient outcomes. Clinical practice guidelines, which aim to reduce variability and improve care, have several drawbacks that limit physician adherence. In contrast, standardised clinical assessment and management plans offer a physician-designed approach to promoting standardisation of care that considers individual patient differences, respects physicians' clinical expertise, and keeps pace with rapidly growing medical knowledge. The orthopaedic community can combine expertise and experiences to enrich educational content and promote best practices through international collaboration. Furthermore, it can be an opportunity to explore new common challenges and innovative solutions, seeking a more comprehensive understanding of sports injuries and their management.^9^, ^10^, ^11^, ^12^

This review examined the relevance of peculiar formations on sports injuries among orthopaedic surgeons. It analysed the current status of formation programs on sports injuries worldwide, assessing the efficacy of existing educational courses. It also focused on successful international collaborations, resulting in improved clinical practices. The study recommends ways to enhance sports injury education through collaborative projects. This could be achieved through networks and global partnerships, leading to the development of more effective and clinically relevant educational models that significantly improve the management of sports injuries in Orthopaedics.

## 2 Methods

All clinical studies addressing educational models, training programmes, or teaching innovations related to orthopaedic sports injury management were considered for inclusion. Only articles published in peer-reviewed journals were selected. Studies in English, German, Italian, French, and Spanish were deemed eligible. Studies classified as level I to IV evidence, according to the Oxford Centre for Evidence-Based Medicine,^13^ were included. The following search framework was defined: (1) Subject: orthopaedic education in sports injury management; (2) Intervention: educational programmes, fellowships, simulation, digital learning, and international training models; (3) Outcomes: structure, implementation, clinical impact, and educational efficacy. In March 2025, a comprehensive search was conducted in PubMed, Web of Science, and Embase. No restrictions were applied regarding the publication year. Medical Subject Headings (MeSH) and search strings are reported in Table 1.

**Table 1:** Medical Subject Headings (MeSH) and search strings used in each database.

Database	MeSH
PubMed	("Sports Injuries"\[MeSH] OR "Athletic Injuries"\[MeSH]) AND ("Education, Medical"\[MeSH] OR "Clinical Competence"\[MeSH] OR "Orthopedic Procedures"\[MeSH]) AND ("Simulation Training"\[MeSH] OR "Fellowships and Scholarships"\[MeSH] OR "Curriculum"\[MeSH]) |
Embase	('sports injury'/exp OR 'athletic injury'/exp) AND ('medical education'/exp OR 'clinical competence'/exp OR 'orthopaedic surgery'/exp) AND ('simulation training'/exp OR 'fellowship'/exp OR 'curriculum'/exp)
Web of Science	TS=("sports injuries" OR "orthopaedic education" OR "orthopaedic training") AND TS=("simulation" OR "fellowship" OR "curriculum" OR "training programme" OR "competency-based")

### 2.1 Global landscape of sports injury education

Sports trauma refers to acute or chronic injuries that most commonly occur during sports or physical exercise; they are not limited to athletes. Engagement in sports holds substantial positive benefits for public health throughout various stages of life. However, the incidence of sport-related injuries is considerable, particularly among youth and young adults, who are at the highest risk. Usually, sports injuries affect the musculoskeletal system, often leading to extended downtime from training and competition. Given the large population engaged in sports across various age groups, interest in this type of injury and its socio-economic impact is growing. The U.S. Consumer Product Safety Commission's National Electronic Injury Surveillance System (NEISS) reports that over 1.9 million individuals annually experience sports-related injuries treated in emergency departments. Contact sports have the highest rates of injury. Over 22 million athletes between the ages of 5 and 22 suffer sports injuries annually, especially in football, basketball, track and field, boxing and cycling.

Moving upstream to primary prevention and effective diagnosis and treatment of sports injuries is a public health priority with significant implications for reducing the long-term consequences of musculoskeletal injuries.^14^ Different health and sports professionals are involved in the prevention, management, and return-to-sport process, including trainers, coaches, radiologists, orthopaedic surgeons, physiatrists, sports medicine physicians, psychologists, physiotherapists, exercise physiologists, and team doctors. The primary targets for preventing musculoskeletal injuries in sports include neuromuscular training and equipment recommendations. In detail, neuromuscular control should be considered, especially in younger athletes undergoing rapid growth and physical development, as its use significantly reduces the risk of musculoskeletal injury. Different training programs have been developed for various sports, and specific neuromuscular strengthening programs are more effective than regular sport-specific training for prevention. Similarly, timely diagnosis and appropriate conservative or surgical treatment make the difference in this context. Various definitions of return to sport (RTS) have been proposed in the past. Recently, a consensus statement defined an RTS continuum: return to participation, RTS, and return to performance. The rehabilitation and RTS program is designed to meet the individual patient's needs, depending on the type and severity of the injury. The active involvement of the patient and family is also essential in the success of the program. Even after proper diagnosis, treatment, and rehabilitation, a sports injury increases the patient's risk of future injury. Educating athletes who have suffered sports injuries is essential to help them avoid further damage, emphasising the importance of pre-season conditioning, sport-specific training and appropriate gear.

In this context, the various training systems used globally should also be considered for educating physicians and other health professionals about sports injuries. Defining clear, practical, consistent, operational criteria to describe an injury that can be applied across different sports is essential for researchers in sports. Still, systems exist to classify injuries based on exposure time, affected tissue, and the severity of the injury. Furthermore, sports injuries vary based on geographical, social, and cultural factors, as different sports are practised depending on location, gender, economic resources, and historical interest.

Distance learning within an electronic learning (e-learning) environment has become a standard practice. The skills acquired and the emerging opportunities significantly influence education in the post-COVID era. A Cochrane review found no difference between traditional and electronic learning in health professionals regarding behaviour, skills or knowledge. Global perspectives on sports injury education in orthopaedics are required to enhance clinical practice. Innovative learning methods, especially augmented reality (AR) and virtual reality (VR) solutions, are emerging worldwide that could help achieve this goal.

### 2.2 Innovative curricular approaches and best practices

Sports injuries pose a significant challenge for healthcare systems worldwide. They may lead to short- and long-term disabilities that have a catastrophic impact on patients and their families. Furthermore, they significantly impact society in terms of economic costs.^15^^,^^16^ Considering that these conditions are constantly increasing over time, all the involved professional figures (such as physicians, physical therapists, orthopaedic surgeons, trainers, and other practitioners) require appropriate and modern training focused on preventing and managing injuries.^17^^,^^18^ Further education regarding sports injuries is essential in both sports and orthopaedic courses.^19^^,^^20^ A comprehensive approach to these pathological situations can improve patients' conditions and enhance research and clinical practice methods.

Many countries worldwide have made significant efforts to incorporate sports injury knowledge into their existing orthopaedic training programs.^21^ Problem-based learning (PBL) is an educational approach in which students learn by actively engaging with fundamental themes and cases, rather than passively receiving knowledge from lectures. This practical problem-solving learning method has several advantages, including easier knowledge acquisition and retention, enhanced active problem-solving ability, increased learning interest, improved communication, teamwork, and critical evaluation skills. Conversely, basic knowledge and theory may be less comprehensive than conventional approaches. Its application in orthopaedic training has been reported as an essential method in China for nurses and residents, as it involves complex cases that can enhance clinical approach and decision-making for specific orthopaedic pathologies. A similar approach is Team-Based Learning (TBL), a structured learning system centred on strategic collaboration and working in teams to solve challenges, find solutions, and apply knowledge. Cremerius et al.,^22^ in their prospective randomised trial on basic musculoskeletal ultrasound skills in 88 students, divided the trainees into three groups (19 team-based learning, 36 peer-assisted learning, and 33 conventional teaching) and showed a significant superiority of the team-based learning group compared to the others. Furthermore, a training program based on acquired skills is a new and innovative method that may address the limitations of traditional time-based training systems. Recently, Nousiainen et al.^23^ reported excellent results after their eight-year experience in a residency training program in orthopaedic surgery at the University of Toronto. The authors emphasised the role of the Competency-Based Curriculum (CBC), an educational system based on confirming trainees’ complete, precise skills and abilities rather than spending a fixed amount of time in training. The CBC allows residents to progress once they demonstrate the required skills and competencies, rather than advancing based solely on their duration in the program. “Hands-on” education programs are efficient in terms of rapid information learning and strong memorisation skills. They provide invaluable real-time experience. This method has been reported to be very effective, especially for learning physical therapy, surgical skills, or sports ultrasound (US).^24^ Hands-on surgical workshops using 3-dimensional models or cadaveric specimens enable trainees to practice delicate surgeries, such as meniscus repair, ACL reconstruction, cartilage restoration, tendon suture, fracture fixation, and other procedures under expert supervision and without the risk of harming patients.^25^^,^^26^ These methods and precise lectures improve knowledge, memory, surgical skills, and confidence.^27^ For some procedures widely used in sports injury management, such as arthrocentesis (a medical procedure that involves a needle puncture into a joint space to remove fluid), the hands-on approach, together with other learning activities, is appropriate to increase the ability and self-confidence of medical students. Furthermore, it reduces the risk for patients.

Following the COVID-19 pandemic, on-site education transitioned to virtual learning in many fields, including orthopaedics and sports medicine.^28^^,^^29^ Other areas in which the hands-on approach seems almost mandatory, such as physiotherapy, have also undergone this change. Some virtual platforms facilitate continual theoretical instruction and emulate hands-on training with live feedback. Virtual education enables global access, encourages collaboration among distant experts, and reduces travel costs and time away from work. Traditional lectures and live demonstrations have been replaced by e-learning and instructional videos, and a survey of 46 students yielded positive results.^30^ Given its effectiveness in the educational setting, this new program has been kept after pandemic restrictions. COVID-19 has demonstrated that virtual didactic, surgical education, and training (and telehealth) are playing a more significant role in recent years, while maintaining the element of human interaction and connecting with patients and trainees at a time when social distancing can be challenging.

Simulation training is a method of learning skills without practising on a live patient, thereby preventing avoidable damage to patients.^31^ Some authors have reported that this training activity should be mandatory in every surgical training program.^31^ Conversely, others question the sustainability and capability of orthopaedic surgery residents to transfer the skills learned in simulation to clinical practice. The Oregon Health & Science University Orthopaedic education program has established a residency curricular model integrated with simulation that can serve as an example for other institutions and programs.^32^ However, some essential characteristics are necessary to be meaningfully integrated into surgical education: feasibility, cost-effectiveness, well-defined goals, and measurable outcomes. These include the assessment of trainees' problem-solving and decision-making skills, the possibility of remediation for poor performance, standardisation in curriculum and board certification, quality control, and avoidance of redundancy.^33^ Virtual reality (VR) and augmented reality (AR) have found numerous applications in orthopaedic surgery and sports medicine over the past few years, aiding in intraoperative procedures and surgical education. A recent systematic review of 57 studies conducted since 2014 found that VR simulation, which provides immersive and interactive training environments that simulate real-life movement patterns, allows unrestricted practice and performance analysis without the need for a mentor, and enhances athletic performance and injury recovery. Ten studies demonstrated its benefits in this area. Furthermore, the possibility of tracking progress makes it a promising tool in sports medicine and injury prevention.^34^ Artificial intelligence (AI) has been increasingly used in orthopaedic settings recently. According to a systematic review of 58 studies covering 12 team sports, it can enhance injury prevention and performance optimisation in team sports.^35^ Multidisciplinary approaches and teams comprising various professionals can be key to comprehensively managing sports injuries.^36^^,^^37^ Interdisciplinary approaches with teamwork and multidisciplinary training sessions (integrating orthopaedic surgeons, physiotherapists, sports psychologists, trainers, and other professionals) are necessary to ensure comprehensive patient care, avoid complications, improve communication, and allow the return to sport. Furthermore, international partnerships offer incredible opportunities to share different methods, become aware of various kinds of expertise, implement the best approaches, and adopt pioneering training systems.^38^^,^^39^ International meetings and consensus can improve patient care and enhance educational standards. A 2016 Qatar conference titled "*Monitoring Athlete Training Loads – The Hows and the Whys*" reunited worldwide professionals to debate modern methods and potential directions in load monitoring. This consensus statement provides a basis for coaches, sports scientists, physicians, and medical professionals, outlining what athlete-load monitoring is, how it can be used in research and practice, why it is essential, and its future applications in sports performance and injury prevention. The PAASS (Pain, Ankle impairments, Athlete perception, Sensorimotor control, Sports performance) framework was established through an international, multidisciplinary consensus to guide return-to-sport decisions after acute lateral ankle sprain injuries. This study involved 155 health professionals working in elite field and court sports and represents a globally accepted approach to standardising return-to-sport decisions, ensuring consistent assessment criteria across different countries and sports disciplines.^40^ International congresses and teams, such as the International Society of Arthroscopy, Knee Surgery, and Orthopaedic Sports Medicine (ISAKOS) Upper Extremity Committee, facilitate cross-border research on injury prevention, consensus, and treatment outcomes, leading to evidence-based practices and standardised management.^41^ International fellowship and exchange programs allow professionals to experience different realities and clinical practices. Programs such as the American Orthopaedic Association-Japanese Orthopaedic Association (AOA-JOA) and the ESSKA (European Society of Sports Traumatology, Knee Surgery & Arthroscopy) Fellowships offer opportunities for young surgeons to train at leading institutions worldwide, promoting diverse perspectives and enriching clinical practice.^42^, ^43^, ^44^ Sports injury education in orthopaedics programs needs international and collaborative approaches characterised by modern methodologies to improve patient outcomes, standardise education and care, and advance the field of sports injury management.

### 2.3 Challenges and barriers in a global context

The variability in healthcare infrastructures is one of the most critical challenges in patient care, research, and global sports injury education. High-income nations typically have well-developed healthcare systems with access to sophisticated diagnostic tools, surgical technologies, and rehabilitation services. Conversely, low- and middle-income countries often face significant challenges in accessing medical resources, trained professionals, and financing for medical education. Addressing barriers and disparities between countries and regions in terms of their impact on healthcare outcomes is crucial. Bouma et al.^45^ have reported that healthcare professionals and their patients face many challenges and barriers. The deficiency of organisation, coordination, and communication between different healthcare workers demonstrates poor interdisciplinary collaboration, which needs to be addressed. Inadequate teaching regarding the best treatment and strategies can limit the outcomes. This last aspect may result from the healthcare provider's lack of knowledge, skills, education, or time. All these difficulties emphasise the need for improved collaboration, adequate training, and education for healthcare providers and patients. To limit challenges and barriers, educating patients can be as important as training healthcare professionals. Indeed, patients' refusal to accept lifestyle interventions or other proposed treatments for various reasons (scepticism, laziness, low motivation, and unwillingness) presents another challenge that hampers the efficacy of therapies. Similarly, patients sometimes struggle to comprehend, realise, or follow instructions.

The availability of new technologies also plays a crucial role in disparities. Several key barriers hinder the equitable implementation of new technologies that benefit patients, healthcare practitioners, and learners in hospitals. These barriers include high costs, inadequate coverage, financial constraints, and insufficient funding, all of which contribute to resistance against adopting new technologies. Furthermore, competition from other firms and less innovative alternatives may impede the acquisition of these technologies. Logistical issues, problems with availability and ordering, and a lack of perceived need are additional factors that may contribute to inequality in the development of new technologies. Bakhshaie et al.^46^ studied healthcare providers' perceptions of barriers in the orthopaedic setting, identifying disparities at various levels that can be categorised as individual, relationship-based and social. The main factors include low health literacy, language barriers, mental health issues, substance use, limited access to technology, weak social support networks, transportation difficulties, job insecurity, insurance gaps, and cultural differences. Branstetter et al.,^47^ discussed how developing technologies are transforming orthopaedic surgical training and approaches to sports injuries. According to the authors, these innovations help address gaps in residency education; however, they also introduce significant challenges. Firstly, there are some ethical issues regarding the assessment of technologies in conjunction with traditional practices: responsible use is necessary, and established, safe methods must not be overlooked. Disparities in access to technology can create inequalities in outcomes among different structures. Furthermore, there is a consistent risk of over-dependence on these tools. Universal guidelines and thoughtful approaches are necessary to maximise the advantages while preserving proficiency.

Vast differences in training between practitioners are another challenging aspect that can create variances among countries or hospitals. Tompson et al.^48^ conducted a study on variability in training experience among first-year orthopaedic surgery residents (PGY-1). The authors conducted this study using a survey of program directors from 79 orthopaedic residency programs. Common non-orthopaedic rotations include general surgery, trauma, intensive care units, and plastic/burn surgery; 91 % of programs implement a formal surgical skills curriculum, often using cadaveric specimens and sawbones. However, the use of simulation technology and other approaches to skills training differs widely among programs. This study demonstrated the elasticity allowed in residency programs and, at the same time, emphasised the necessity of standardised guidelines to guarantee homogeneous expertise across programs.

Sports injury management diverges significantly in terms of orthopaedic training, duration, and certification standards, which vary substantially between countries. Sobel et al.^49^ evaluated worldwide variability in orthopaedic surgery training by analysing educational pathways across 11 countries. The authors highlighted differences in pre-medical education, medical school duration, residency structure, and specialisation requirements. They emphasised the lack of global standardisation in orthopaedic training, noting that residency lengths range from three to six years, medical school durations vary from five to seven years, and some countries require additional public service commitments. All these differences underscore the necessity of improving training models and ensuring equal competency among orthopaedic surgeons worldwide. A comprehensive approach is essential to address disparities in education, training, and healthcare infrastructure. Increasing services and collaborations can be crucial to bridging the gaps between different realities and enhancing healthcare resources. Standardising education and training methods through international cooperation can assist professionals in adopting innovative approaches. Effective cost management and equitable allocation of resources can help mitigate disparities among institutions and countries. Expanding the use of new technologies can be advantageous, particularly in low- and middle-income countries; however, a judicious application of these technologies is always advisable to maintain a balance between technological advancements and traditional surgical skills. Developing universal guidelines and fostering universal collaboration can create a more standardised, equitable, and practical approach to orthopaedic surgery, sports, and education related to these fields.

## 3 Conclusions

Sports injuries are a growing global challenge with clinical, educational, and economic impact. This review highlights the need for comprehensive, standardised training in orthopaedic sports medicine to improve prevention, diagnosis, and treatment. Integrating simulation, competency-based training, and digital tools into curricula, alongside international cooperation and sharing of best practices, can reduce disparities, standardise care, and enhance outcomes worldwide.

## Consent to participate

Not applicable.

## Consent to publish

Not applicable.

## Authors’ contribution

FM: conception and design, drafting (original and revision); LL, MM: supervision, drafting (revision); TB: literature search; LP; RJ: supervision; All authors have agreed to the final version to be published.

## Ethical approval

This study complies with ethical standards.

## Registration and protocol

The present study was not registered.

## Availability of data and materials

The datasets generated during and/or analysed during the current study are available throughout the manuscript.

## Funding

The authors received no financial support for the research, authorship, and/or publication of this article.

## Competing interests

The authors declare that they have no competing interests for this article.
